# The Influence of Noise Perception and Parent-Rated Developmental Characteristics on White Noise Benefits in Children

**DOI:** 10.3390/jemr19010018

**Published:** 2026-02-05

**Authors:** Erica Jostrup, Marcus Nyström, Göran B. W. Söderlund, Emma Claesdotter-Knutsson, Peik Gustafsson, Pia Tallberg

**Affiliations:** 1 Child and Adolescent Psychiatry, Department of Clinical Sciences, Lund University, 221 84 Lund, Swedenpeik.gustafsson@med.lu.se (P.G.);; 2 Humanities Lab, Lund University, 221 00 Lund, Sweden; 3 Faculty of Teacher Education Arts and Sports, Western Norway University of Applied Sciences, Campus Sogndal, 6856 Sogndal, Norway; goran.soderlund@hvl.no; 4 Outpatient Department, Child and Adolescent Psychiatry Clinic, Region Skåne, 221 85 Lund, Sweden

**Keywords:** white noise, child development, noise perception, memory-guided saccade, prolonged fixation

## Abstract

White noise has been proposed to enhance cognitive performance in children with ADHD, but findings are inconsistent, and benefits vary across tasks and individuals. Such variability suggests that diagnostic comparisons may overlook meaningful developmental differences. This exploratory study examined whether developmental characteristics and subjective evaluations of auditory and visual white noise predicted performance changes in two eye-movement tasks: Prolonged Fixation (PF) and Memory-Guided Saccades (MGS). Children with varying degrees of ADHD symptoms completed both tasks under noise and no-noise conditions, and noise benefit scores were calculated as the performance difference between conditions. Overall, white-noise effects were small and dependent on noise modality and task. In the PF task, large parent-rated perceptual difficulties and high visual noise discomfort were associated with improved performance under noise. In the MGS task, poor motor skills predicted visual noise benefit, whereas large visual noise discomfort predicted reduced noise benefit. These findings suggest that beneficial effects of white noise are influenced by developmental characteristics and subjective perception in task-dependent ways. The results highlight the need for individualized, transdiagnostic approaches in future noise research and challenge the notion of white noise as categorically beneficial for ADHD.

## 1. Introduction

White noise stimulation refers to the presentation of a constant, random sensory (auditory, visual, tactile or electrical) signal that contains equal intensity across a wide range of frequencies [1]. In auditory form, it resembles a steady “shh” sound, similar to static or a detuned radio. In its visual form, it typically appears as a rapidly flickering pattern of randomly distributed black and white pixels.

Recent reviews suggest that white noise stimulation may enhance cognitive performance in children with ADHD [2,3]. One proposed mechanism for this effect is stochastic resonance, a phenomenon in which the addition of random noise enhances the detection or processing of weak signals in non-linear systems [4,5]. However, this hypothesis has been met with criticism, as empirical findings are inconsistent and sometimes contradictory [6,7,8,9,10,11,12,13,14,15,16,17]. As an alternative to stochastic resonance, white noise effects have also been attributed to mechanisms such as auditory masking [8] or increased arousal [18], but neither explanation alone accounts for the heterogeneous and modality-spanning benefits reported in the literature.

White noise has been reported to improve various aspects of executive functioning, including visuospatial and verbal working memory, response time variability, and response inhibition [6,10,11,15,16]. Improvements in academic outcomes, such as reading and writing speed, and reductions in off-task, hyperactive, or impulsive behaviors, have also been documented [7,8,9,14]. However, findings across studies are mixed, and the mechanisms underlying the so-called noise benefit remain unclear. For instance, Jostrup, Claesdotter-Knutsson [12] found no positive effects of auditory white noise on cognitive performance in children with ADHD. Similarly, earlier studies using the same cognitive task (i.e., the Spanboard task) have reported inconsistent results [11,13,15,16]. These studies applied group-level comparisons between clinical and neurotypical samples. Importantly, even when executive function tests reliably distinguish children with ADHD from neurotypical controls at the group level, effect sizes are typically modest, and many individuals with ADHD do not exhibit clear neurocognitive impairments [19]. Moreover, executive function measures show limited diagnostic specificity [20,21,22], as similar patterns of dysfunction are observed across a range of developmental and psychiatric conditions [23,24,25]. Such findings highlight the heterogeneity within diagnostic categories and underscore the limitations of relying solely on diagnostic group comparisons. Recent frameworks instead emphasize a transdiagnostic perspective, viewing difficulties in attention, perception, motor control, and learning as dimensions that cut across traditional diagnostic boundaries [26,27,28]. This perspective resonates with neurodiversity approaches, which highlight the wide range of individual differences within neurodevelopmental conditions [29].

Beyond ADHD, many children experience developmental challenges, such as motor coordination problems, language or learning difficulties, or atypical sensory processing [26]. These characteristics may influence how they respond to external stimulation. Nevertheless, research on white noise has rarely examined these broader developmental profiles. Notably, beneficial effects of white noise have been observed in a wide range of developmental domains in neurotypical populations. For instance, studies in adults without psychiatric diagnoses have reported improvements in word learning, long-term recall, and auditory working memory [30,31,32,33]. Additional benefits have been observed for spatial navigation [34], perceptual judgments [35], and multimodal sensorimotor performance [36]. However, these effects are not universal; in some studies, no group-level effects emerged, and only subsets of individuals benefited [37,38].

Another potential factor underlying such variability in findings, apart from individual developmental profile, is noise perception. Noise perception refers to the individual’s subjective experience and evaluation of noise, including how loud, distracting, or pleasant it feels. Two people exposed to the same stimulus may perceive it very differently: one may find it neutral or even helpful, while another experiences it as highly unpleasant. Noise perception is conceptually distinct from noise sensitivity. Noise sensitivity refers to a more stable trait reflecting how reactive a person generally is to auditory or sensory input.

Research outside the white-noise field shows that subjective noise perception can substantially influence cognitive and emotional outcomes. For example, individuals with high noise sensitivity perform worse on memory and arithmetic tasks under moderate traffic noise [39] and report higher stress and annoyance even at identical decibel levels [40]. Similarly, soundscape research demonstrates that sounds appraised as more pleasant or congruent with context cause less distraction than unpleasant sounds of the same intensity. While chronic environmental noise exposure typically impairs performance and well-being [41,42,43], controlled laboratory studies indicate that transient, task-concurrent stimulation (such as white noise) can sometimes enhance focus or alertness [44].

Taken together, these findings suggest that how noise is perceived may be just as important as the physical properties of the noise itself in determining its cognitive effects.

Despite the growing literature on white noise and cognitive performance, few studies have systematically examined how children’s developmental profiles or their subjective experiences of noise relate to objective performance changes under noise stimulation. This gap is particularly relevant given the marked inconsistency in reported white noise effects across studies. These mixed findings suggest that diagnostic group comparisons alone may be insufficient to explain variability in noise-related performance outcomes. Instead, individual developmental characteristics and subjective noise perception may moderate how external stimulation influences task performance, helping to explain why identical noise can facilitate performance in some individuals but hinder it in others.

To address this gap, the present study explores the relationship between noise perception, developmental characteristics, and task performance in two eye-movement paradigms. To ensure that a broad spectrum of individual developmental profiles was covered, both children with and without an ADHD diagnosis were recruited for the study. Moving beyond diagnostic categories, we here focus on investigating whether individual differences in parent-rated developmental functioning (5–15R domains) and children’s subjective experience of noise are associated with noise benefit. Specifically, we examine auditory and visual white noise benefit during a Prolonged Fixation and a Memory-Guided Saccade task. By examining both sensory and cognitive aspects of performance, this study aims to contribute preliminary exploratory insight into for whom and under what conditions white noise may influence cognitive and oculomotor functioning.

## 2. Methods

Data for this study were collected as part of a larger study; only relevant parts of the method are described here. For details, we refer the reader to the publication from Jostrup, Claesdotter-Knutsson [12].

### 2.1. Study Design

The study employed a within-subject experimental design in which participants completed two oculomotor tasks: a Memory-Guided Saccade (MGS) task and a Prolonged Fixation (PF) task. Each task was performed under four noise conditions: a silent (no-noise) condition, an auditory white noise condition, and two conditions with varying levels of visual white pixel noise. The within-subject manipulation of noise conditions was used to derive individual noise benefit scores, reflecting performance differences between noise and no-noise conditions for each task and noise modality. These noise benefit scores served as the primary outcome measures in subsequent analyses examining individual differences in noise benefit.

The present manuscript focused on individual variability in noise effects rather than on formal comparisons between experimental conditions. Group- and condition-level analyses based on the same experimental design have been reported previously [12]. The PsychoPy code used to run the experiment is publicly available on GitHub: https://github.com/marcus-nystrom/white-noise-exp (commit 48607f48, accessed on 6 March 2024).

### 2.2. Participants

The study included 97 participants aged from 7 to 16 years: 52 children with an ADHD diagnosis (31 boys, 21 girls; M = 11.7 years, SD = 1.7) and 45 typically developing children (TDC; 20 boys, 25 girls; M = 11.7 years, SD = 2.4). Children in the ADHD group were recruited from the outpatient child and adolescent psychiatry clinic in Lund, Sweden, while TDC were recruited from local schools in the same region.

To capture a naturalistic and representative sample reflecting the diversity within each group [45], exclusion criteria were kept to a minimum. For the TDC group, inclusion was limited to the 7–16-year age range, and exclusion criteria comprised any diagnosed neurodevelopmental disorder. Children in the ADHD group were diagnosed according to DSM-5-TR criteria [46], encompassing combined, predominantly inattentive, and predominantly hyperactive-impulsive presentations. All diagnoses were confirmed by senior child and adolescent psychiatry consultants (two of the study’s authors).

Children receiving methylphenidate were asked to suspend medication 24 h prior to participation. Those using other pharmacological treatments or failing to comply with the withdrawal instructions were excluded from the study.

### 2.3. Test Battery

#### 2.3.1. Prolonged Fixation Task

**Figure 1:**
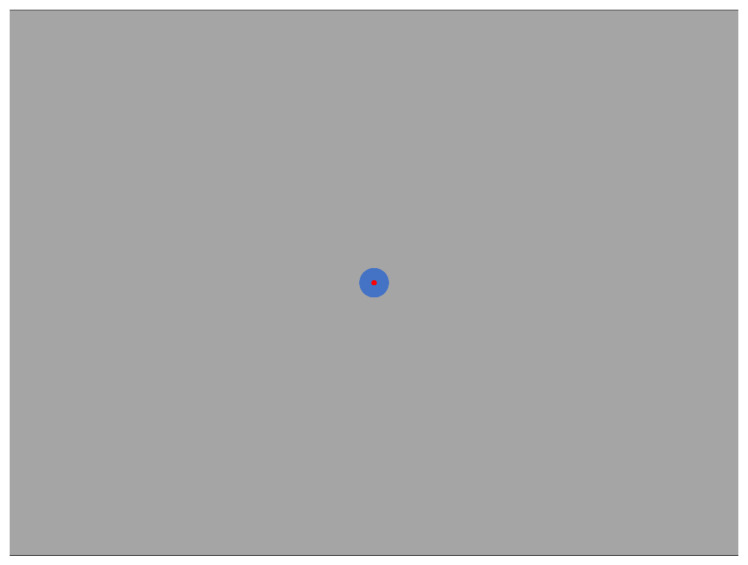
The Prolonged Fixation task required the participants to fixate on a central fixation point for 60 s.

In the Prolonged Fixation (PF) task, participants were instructed to maintain gaze on a fixation point displayed at the center of the screen for a duration of 60 s. The fixation stimulus consisted of a blue disk (1° visual angle) with a smaller red disk (0.2°) centered within it (see Figure 1).

#### 2.3.2. Memory-Guided Saccade Task

In the Memory-Guided Saccade (MGS) task, participants were instructed to fixate on a central point for as long as it remained visible. While fixating, a white disk briefly appeared in one of four possible peripheral locations. Participants were instructed not to look directly at the disk but to remember its position. Once the central fixation point disappeared, participants were to immediately shift their gaze to the remembered location. The disc then reappeared at its original location, allowing for a corrective saccade if necessary.

The central fixation point was identical to the one used in the PF task—a blue disk (1° visual angle) with a red disk (0.2°) at its center. Following previous MGS paradigms [47,48], the central fixation point remained visible for a randomized duration between 2000 and 3500 ms before the peripheral disk appeared and for another 2000 to 3500 ms after the disk disappeared.

**Figure 2:**
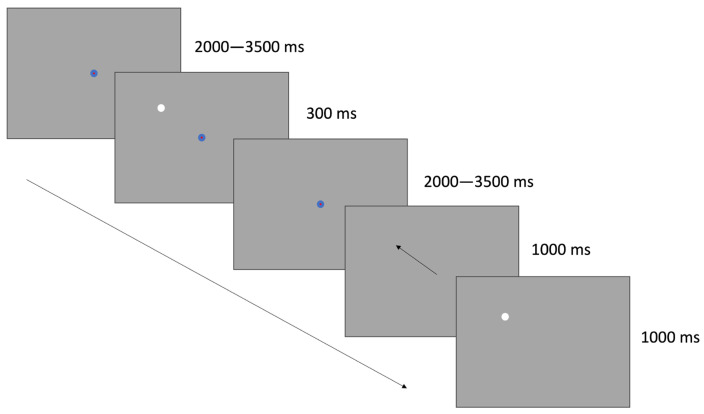
In the MGS task, participants fixated on a central point until it disappeared. Meanwhile, a white disk appeared in one of four directions, to which participants were instructed not to shift their gaze but to remember its location. Upon disappearance of the fixation point, participants were prompted to quickly redirect their gaze to the remembered location of the disk. Subsequently, the disk reappeared, allowing participants to correct any possible deviation between the gaze location and the disk location. The arrow illustrates the timeline of the illustrations.

The peripheral white disk (1°) was presented at one of four possible directions (45°, 135°, 225°, or 315°), each located 10° from the central fixation point, and was visible for 300 ms. The disk reappeared 1000 ms after the central fixation point was turned off and remained visible for 1000 ms to enable corrective saccades (see Figure 2). Each participant completed 30 MGS trials per noise condition.

### 2.4. White Noise Stimulation

#### 2.4.1. Auditory White Noise

Auditory white noise was generated using a uniform distribution (U [0, 255]) and presented as a stereo signal, with independent arrays created for the left and right channels. The audio was sampled at 48,000 Hz, digitized at 16-bit resolution, and saved in uncompressed .wav format. Prior to each experimental session, the sound level was calibrated to 78 dB SPL using a UNI-T UT351/352 sound level meter (Uni-Trend Technology, Dongguan, China). Calibration was conducted separately for each computer and earphone combination to ensure consistent presentation across participants.

#### 2.4.2. Visual White Pixel Noise

**Figure 3:**
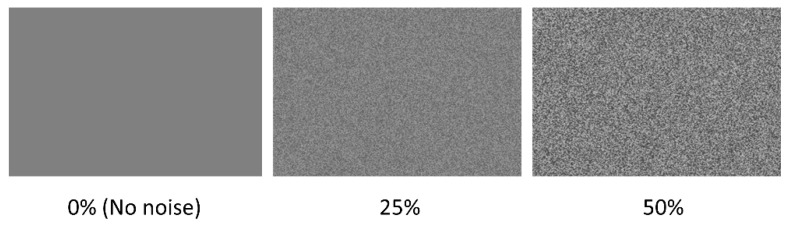
The visual white pixel noise at 0% (no noise), 25% and 50%.

Visual white noise was applied to each pixel of the stimulus image by blending it with a transparent noise layer of the same dimensions, with pixel values sampled from a uniform distribution (U [0, 255]), where 0 corresponds to black and 255 to white. The intensity of the noise was manipulated through the transparency of the noise layer: 0% transparency produced no visible noise, while 100% resulted in full occlusion by noise. In this study, transparency levels of 25% and 50% were used (see Figure 3). The noise image was refreshed on every screen update (60 Hz) to create dynamic visual noise throughout stimulus presentation.

### 2.5. Assessments

#### 2.5.1. Noise Rating

Participants completed a post-task questionnaire consisting of five items, each rated on a 10 cm visual analog scale (VAS). The first two items assessed perceived task difficulty under auditory and visual noise conditions, respectively, with anchors ranging from ‘easier’ to ‘harder’. The third and fourth items measured discomfort associated with the noise interventions, phrased as “Did you feel any discomfort when listening to the noise sound?” (anchors: not at all—very unpleasant to listen to) and “Did you feel any discomfort when looking at the pixel noise?” (anchors: not at all—very unpleasant to look at). The fifth item evaluated overall task enjoyment, ranging from ‘fun’ to ‘boring’. Participants indicated their response by marking a point along each line, which was subsequently measured in millimeters from the left anchor to yield a score between 0 and 100, with higher values reflecting greater difficulty, greater discomfort, and lower enjoyment.

#### 2.5.2. 5–5R

The 5–15R [49,50] is a standardized assessment tool consisting of 181 items that reflect everyday situations, covering domains such as motor skills, executive functioning, memory, and perception. It is designed for children aged 5 to 17 years and is commonly used to identify behavioral difficulties and potential developmental disorders [50]. For each item, respondents rate the extent to which the behavior applies using a four-point scale: “no,” “a little,” “a great deal,” or “very much.” We applied the composites assessing functions corresponding to Neurodevelopmental Disorders (NDDs) (APA, DSM-5) (i.e., Intellectual Disabilities, Communication Disorders, Autism Spectrum Disorders, ADHD, Specific Learning Disorders, and Motor Disorders): Motor skills, Executive Functions, Perception, Memory, Language and Communication, Learning Skills, and Social Skills.

### 2.6. Procedure

Prior to the start of the experiment, participants were introduced to the different noise conditions and received standardized instructions on how to perform the Memory-Guided Saccade (MGS) task, following procedures similar to those described by Mahone, Mostofsky [48]. They were instructed, “As long as the center fixation point is visible, keep your gaze fixed at the center. Do not look at the flash when it appears but remember its location. When the center point disappears, immediately move your gaze to the place where you saw the flash.”

To ensure task comprehension, participants completed a training session for the MGS task. The training assessed performance on a trial-by-trial basis, and participants were required to achieve at least three correct responses out of five to proceed. A correct trial was defined as maintaining gaze within 6° of the central fixation point until its disappearance, followed by a saccade to within 6° of the flash location. The training was conducted without any noise stimulation.

Following successful training, participants completed a total of eight tasks: two initial MGS tasks, four Prolonged Fixation (PF) tasks, and two final MGS tasks. Each task was completed under one of four noise conditions: no noise, auditory white noise, visual white pixel noise at 25%, and visual white pixel noise at 50%. The order of noise conditions was randomized across tasks for each participant, ensuring exposure to all conditions.

A planned break was provided between the initial MGS and PF tasks, during which participants received instructions for the PF task. A second break was given between the PF and final MGS tasks, allowing time for rest and refreshments. The total duration of the training and experimental session was approximately 50 min.

Upon completing the experimental tasks, participants completed a noise-rating questionnaire to assess their subjective experience of the tasks and the noise conditions. In addition, legal guardians completed the 5–15R form to provide standardized assessments of the children’s behavioral and developmental profiles.

### 2.7. Data Collection and Processing

Data collection was initiated in October 2023 and ended in February 2024. Binocular eye movements were recorded at 600 Hz using Tobii Pro Spectrum eye trackers (Tobii AB, Stockholm, Sweden). Calibration and stimulus presentation were conducted using PsychoPy and Titta [51], following the same procedures described in detail by Jostrup, Nyström [52]. Participants were seated with head support at a fixed distance from the monitor in a controlled lab environment. All sessions used similar lighting and auditory equipment.

### 2.8. Data Analysis

Eye movement data were preprocessed and analyzed using the I2MC algorithm [53], with fixations, saccades, and data quality metrics computed as described in Jostrup, Claesdotter-Knutsson [12]. Separate analyses were conducted for the PF and MGS tasks.

For PF, the number of intrusive saccades was calculated, as this variable has shown the largest effect sizes in differentiating between children with and without ADHD in previous publications [54]. Saccades with amplitudes ≥ 2° were classified as intrusive saccades, in accordance with previous definitions [55]. Saccades occurring at the beginning of the task that served to direct gaze toward the central fixation point were excluded from the analysis. For MGS, we analyzed the number of anticipatory saccades, which also has been suggested as the measure that separates children with and without ADHD with the largest effect sizes [54]. Anticipatory saccades were defined as saccades initiated before the offset of the central fixation point, including those occurring within 80 ms after fixation offset, as this interval is shorter than the minimum time required to react to the disappearance of the fixation point. Anticipatory saccades were identified as saccades with amplitudes ≥ 2°.

A noise benefit variable was calculated as the difference in performance between the noise and the no-noise conditions. For the visual modality, the two noise levels were averaged since previous studies have not observed differential effects of noise intensity [12]. Separate noise benefit scores were thus derived for visual and auditory noise. Analyses based on individual visual noise levels (25% and 50%) are provided in the Supplementary Material (Supplementary Tables S5–S8).

Trials with >20% data loss from both eyes were excluded from the analysis. In total, 14 trials (4%) from 8 participants were excluded from the PF task. In the MGS task, trials with >20% data loss from both eyes between flash onset and reappearance of the flash were excluded. A total of 347 trials (3%) from 46 participants were excluded from the MGS task.

### 2.9. Statistical Analysis

All statistical analyses were performed in R [56], version 4.4.3. Because this study was exploratory, the aim was not to identify strong associations but to examine whether consistent tendencies could be observed linking task performance under noise to subjective experiences of noise and/or developmental characteristics (5–15R). As such, individual predictors were reported descriptively even in models with limited explained variance.

#### Regression Analyses

For each noise modality (visual and auditory) and task (PF and MGS), a multiple linear regression model was fitted with performance (noise benefit score) as the dependent variable. Independent variables were subjective ratings of task difficulty and noise discomfort as well as four domains of the 5–15 scale (motor skills, executive functions, perception, and learning). Due to high collinearity among 5–15 scale composites, we selected this subset for analysis based on their theoretical relevance to noise interference and cognitive task regulation. However, zero-order (bivariate) correlations between performance measures (PF and MGS) and 5–15R domains were computed as a first explorative step; these correlations were generally small and task-dependent, with only a few modest associations observed. Specifically, higher perceived discomfort was associated with greater performance benefit in the PF task, whereas weaker motor skills were associated with greater visual noise benefit in the MGS task. The full correlation matrixes are presented in Supplementary Tables S1–S4. Age and sex were included as covariates, since previous studies on noise have suggested that sex should be considered [57] and since we have a large age span that might affect performance in the PF and MGS tasks.

All variables were inspected for normality and homoscedasticity using residual plots. To address skewness, variables with non-normal distributions were transformed using the Yeo–Johnson transformation, which accommodates both positive and negative values. Specifically, the variables auditory noise benefit and visual noise benefit at 50% visual noise, during the MGS task, were transformed prior to analysis. All variables were standardized (z-scores) prior to regression analysis.

Effect sizes (standardized β coefficients) and 95% confidence intervals were reported for all models. Emphasis was placed on the relative direction and magnitude of effects rather than on statistical significance.

## 3. Results

### 3.1. 5–15R

Before examining whether developmental characteristics predict children’s responsiveness to sensory noise, we first describe the distribution of 5–15R scores for the domains of motor skills, executive functions, perception, and learning in our sample. Because our analyses rely on variability across these domains rather than on diagnostic contrasts, we assessed whether the combined sample provided sufficient breadth and overlap in parent-rated difficulties.

Across the selected 5–15R domains, children with ADHD showed markedly higher parent-rated difficulties than typically developing controls (TDC). Group differences were large and statistically significant for every domain (all *p* ≤ 0.001). The ADHD group scored substantially higher on executive functions (M = 1.25, SD = 0.33) compared to the TDC group (M = 0.17, SD = 0.19), *t*(84) = –19.78. Pronounced differences were also observed for learning skills (ADHD: M = 0.99, SD = 0.47; TDC: M = 0.17, SD = 0.25), *t*(79) = –10.61, motor skills (ADHD: M = 0.49, SD = 0.36; TDC: M = 0.06, SD = 0.12, *t*(62) = −8.13), and perception (ADHD: M = 0.39, SD = 0.27; TDC: M = 0.06, SD = 0.08, *t*(62) = −8.38).

**Figure 4:**
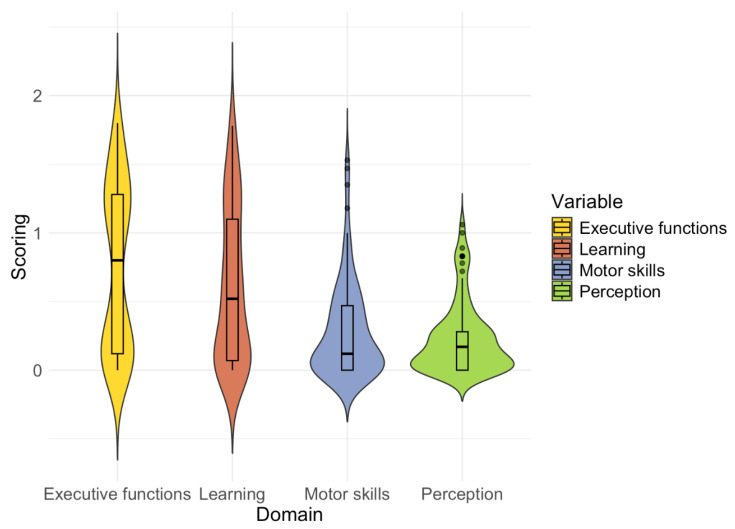
Distribution of 5–15R parent-rated scores in the domains of executive functions, learning, motor skills and perception.

Although these group-level differences were large and consistent across domains, there was also considerable variability within both groups, indicating overlapping distributions of scores (cf. Figure 4). This suggests that while children with ADHD generally showed greater difficulties, individual profiles varied widely, and some children in the control group also displayed elevated scores in specific areas.

### 3.2. Regression Analyses

#### 3.2.1. Prolonged Fixation Task

The regression model examining auditory noise benefit showed that overall, predictors explained little variance in performance. Among the included variables, only the 5–15R perception scale showed a statistically significant positive association with auditory noise benefit (c.f. Table 1), indicating that children with more severe parent-rated perceptual difficulties tended to show slightly better performance improvements under auditory noise for the Prolonged Fixation task.

The regression model for visual noise benefit accounted for a modest proportion of variance in performance (see Table 2). A significant association was found between perceived noise discomfort and visual noise benefit, which indicates that participants who experienced the visual noise as more unpleasant tended to show greater performance improvements when noise was present.

#### 3.2.2. Memory-Guided Saccade Task

The regression model for auditory noise benefit in the MGS task accounted for little variance in performance (c.f. Table 3). None of the predictors reached statistical significance.

The regression model for visual noise benefit in the MGS task explained a small proportion of variance in performance (see Table 4). Two predictors reached statistical significance. Motor skills were significantly associated with visual noise benefit, indicating that children with higher parent-rated motor difficulties tended to show improvements in performance during visual noise stimulation. Perceived noise discomfort was also significantly associated with performance benefit, suggesting that participants who experienced the visual noise as more unpleasant tended to benefit less.

## 4. Discussion

The present exploratory study investigated how individual differences in developmental characteristics and subjective experiences of white noise relate to performance in two oculomotor tasks. Rather than focusing on diagnostic group differences, the aim was to identify patterns of noise responsiveness across cognitive and sensory domains. This was achieved using both objective performance measures and standardized parent ratings on developmental difficulties from the 5–15R questionnaire.

The study revealed small and task-dependent associations between noise-related performance changes and a limited set of subjective and developmental factors. While diagnosis was not examined here, prior analyses using the same sample found no diagnostic differences in noise effects [12]. This implies that individual characteristics and different task demands, rather than diagnostic group, may be more relevant for understanding variability in noise responsiveness.

### 4.1. Prolonged Fixation Task

In the Prolonged Fixation (PF) task, which mainly measures sustained attention, we observed two notable findings. First, children with more severe parent-rated perceptual difficulties tended to show slightly higher performance improvements under auditory noise. One possible explanation for this is that children with weaker sensory discrimination may rely more on external stimulation to maintain arousal or engagement, consistent with arousal-based accounts of stochastic resonance [4].

Second, a strong positive association was found between perceived noise discomfort and visual noise benefit. This indicates that participants who experienced visual noise as more unpleasant tended to show larger performance improvements on the PF task when noise was present. Studies in other sensory domains show similar effects, where unpleasant odors or high-arousing negative stimuli enhance alerting attention or vigilance [58,59,60]. Thus, in simple vigilance tasks like PF, subjective unpleasantness may coincide with a beneficial increase in physiological arousal. This, in turn, may lead to improved performance.

The trend-level association with parent-rated learning difficulties indicates that children with lower learning-related abilities might have been more likely to benefit from visual noise, consistent with the idea that individuals with reduced baseline arousal or weaker regulatory control gain more from additional stimulation [61].

Other developmental characteristics, such as motor skills and executive functions, showed no meaningful associations with PF task performance, suggesting that this task is primarily modulated by sensory and perceptual factors rather than by higher-level cognitive or motor processes.

### 4.2. Memory-Guided Saccade Task

For the MGS task, which places high demands on inhibitory control, visuospatial working memory, and motor coordination, a different pattern emerged. Parent-rated motor skills were significantly associated with visual noise benefit, suggesting that children with weaker parent-rated motor abilities tended to show greater improvements when performing under visual noise. Moreover, perceived noise discomfort was significantly associated with performance benefit for the MGS task, indicating that participants who experienced visual noise as more unpleasant tended to benefit less.

The opposite effects of noise discomfort in PF versus MGS suggest that task demands may shape the direction of noise effects. Since the PF task is a low-demand vigilance task with monotonous fixation, mild aversive or high-arousal stimulation may increase alertness, benefiting performance, even when perceived as unpleasant. On the other hand, the MGS task is a high-demand executive motor task. Here, the subjective perception of an aversive stimulation could contribute to sensory overload, disrupting inhibitory control and motor precision. This task-specific pattern matches theories of moderate brain arousal and noise susceptibility [4,62], where stimulating input is beneficial only when it counteracts under-arousal but becomes disruptive when cognitive load is already high. Alternative mechanisms may also contribute to these effects. For example, visual noise may capture attention or increase sensory load depending on individual sensory tolerance [63,64], thereby contributing to task-specific variability in noise responsiveness.

No associations were observed for auditory noise, suggesting that auditory stimulation does not interact strongly with the visuomotor mechanisms required for anticipatory saccade control.

### 4.3. Noise Perception and Task Performance

Across both tasks, subjective noise perception emerged as an important predictor of noise responsiveness. However, noise perception has rarely been studied in the white noise literature. Importantly, human perception of noise involves psychological factors, such as expectations, appraisal, habituation, and individual differences. These factors mediate noise impacts beyond the physical sound itself. Notably, research outside the white-noise field shows that subjective noise perception can substantially influence cognitive and emotional outcomes. For example, individuals with high noise sensitivity perform worse on memory and arithmetic tasks under moderate traffic noise [39] and report higher stress and annoyance even at identical decibel levels [40]. Similarly, soundscape research demonstrates that sounds appraised as more pleasant or congruent with context cause less distraction than unpleasant sounds of the same intensity. While chronic environmental noise exposure typically impairs performance and well-being [41,42,43], controlled laboratory studies indicate that transient, task-concurrent stimulation (such as white noise) can sometimes enhance focus or alertness [44]. Our results add to this literature by showing that momentary noise discomfort can either improve or impair performance depending on the nature of the task.

### 4.4. Developmental Functioning and Task Performance

Despite including subscales from a broad developmental assessment (5–15R), only sensory-motor domains (perception and motor skills) predicted noise responsiveness in the PF and MGS tasks. Executive functions, memory, and language did not contribute once other factors were included. This aligns with a growing body of research suggesting that noise effects may operate through sensory and arousal regulation mechanisms rather than executive control per se [18,61], even though many studies have measured executive outcomes [6,10,11,15].

Nevertheless, research on white noise has rarely examined these broader developmental profiles. Notably, beneficial effects of white noise have been observed in a wide range of developmental domains in neurotypical populations. For instance, studies in adults without psychiatric diagnoses have reported improvements in word learning, long-term recall, and auditory working memory [30,31,32,33], as well as enhanced spatial navigation [34], accelerated perceptual judgments [35], and improved multimodal sensorimotor performance [36]. However, these effects are not universal; in some studies, no group-level effects emerged, and only subsets of individuals benefited [37,38].

Our study aligns with the broader pattern of heterogeneous and individual-specific effects of white noise. These findings reinforce the idea that white noise effects cannot be understood through diagnostic comparisons alone and that developmental traits and subjective perception likely mediate whether noise helps or hinders performance. The growing body of evidence suggesting that ADHD symptoms and genetic and environmental factors have remained stable although an increase in diagnoses has been seen [65,66] further supports the need for a novel approach to investigating noise benefit.

Although the current analyses were exploratory and the observed effects were small, the findings generate several testable hypotheses. First, that sensory and motor characteristics moderate noise responsiveness. Second, that subjective noise discomfort exerts task-dependent effects—enhancing vigilance but disrupting executive/motor tasks. Finally, we propose that noise perception should be routinely assessed in noise stimulation studies, as it may explain heterogeneity in both clinical and neurotypical populations.

### 4.5. Strengths and Limitations

Strengths of this study include the multimethod design, integration of objective eye-movement measures with subjective noise ratings and parent-rated developmental characteristics, and the use of two oculomotor tasks with distinct cognitive demands. Several limitations should also be acknowledged. The study was explicitly exploratory, and the modest sample size for the regression analyses limited statistical power and the precision of effect estimates, thereby restricting the generalizability of the findings. No corrections for multiple comparisons were applied, increasing the risk for Type I errors, and the relatively low explained variance across models indicates that additional, unmeasured factors likely contribute to individual differences in noise responsiveness. Although stimulant medication was controlled by requiring a washout period prior to testing, residual or long-term effects cannot be entirely ruled out. In addition, subjective ratings of noise and task difficulty were collected only after completion of all experimental tasks, which may have introduced retrospective bias in participants’ evaluation. Moreover, all developmental measures were based on parent ratings, which may not fully capture the cognitive processes engaged during the tasks. While the 5–15R questionnaire is a well-validated instrument, we did not assess sample-specific reliability in the present study, which should be considered when interpreting associations involving parent-rated developmental domains.

## 5. Conclusions

White noise does not exert uniform effects on children’s cognitive performance. Instead, its influence is shaped by the interplay between task demands, sensory-motor developmental characteristics, and subjective noise perception. These findings highlight the need for individualized, transdiagnostic approaches in future noise research and challenge the notion of white noise as categorically beneficial for ADHD.

## Figures and Tables

**Table 1:** Regression results for the number of intrusive saccades in the PF task—auditory noise benefit.

Auditory Noise Benefit	R^2^	Adjusted R^2^	F(8, 80)	*p*
	0.07	−0.02	0.76	0.639
**Predictor**	**β**	**95% CI**	***t*-value**	
Noise discomfort (auditory)	−0.09	[−0.34, 0.16]	−0.71	0.483
Perceived task difficulty (auditory noise)	0.04	[−0.21, 0.30]	0.32	0.747
Motor skills	−0.21	[−0.52, 0.11]	−1.30	0.196
Executive functions	−0.14	[−0.51, 0.24]	−0.72	0.473
Perception	**0.36**	[0.02, 0.70]	**2.13**	**0.037**
Learning	−0.12	[−0.50, 0.26]	−0.62	0.539
Sex (female = 1)	0.13	[−0.34, 0.59]	0.54	0.593
Age	−0.05	[−0.29, 0.19]	−0.38	0.703

Note. β = standardized coefficients. CI = confidence interval. Significant effects (*p* < 0.05) are shown in bold. All predictors were standardized prior to analysis, and variables with skewed distributions were Yeo–Johnson transformed. N = 89.

**Table 2:** Regression results for the number of intrusive saccades in the PF task—visual noise benefit.

Visual Noise Benefit	R^2^	Adjusted R^2^	F(8, 77)	*p*
	0.21	0.13	**2.60**	**0.014**
**Predictor**	**β**	**95% CI**	***t*-value**	
Noise discomfort (visual)	**0.39**	[0.17, 0.61]	**3.47**	**0.001**
Perceived task difficulty (visual noise)	−0.17	[−0.39, 0.04]	−1.60	0.114
Motor skills	−0.05	[−0.35, 0.25]	−0.34	0.735
Executive functions	0.00	[−0.35, 0.35]	−0.00	0.998
Perception	0.17	[−0.16, 0.49]	1.02	0.311
Learning	−0.33	[−0.69, 0.02]	−1.87	0.065
Sex (female = 1)	0.21	[−0.23, 0.64]	0.95	0.344
Age	0.08	[−0.14, 0.31]	0.74	0.462

Note. β values are standardized coefficients. CI = confidence interval. Significant effects (*p* < 0.05) are shown in bold. All predictors were standardized prior to analysis, and variables with skewed distributions were Yeo–Johnson transformed. N = 86.

**Table 3:** Regression results for the number of anticipatory saccades in the MGS task—auditory noise benefit.

Auditory Noise Benefit	R^2^	Adjusted R^2^	F(8, 82)	*p*
	0.06	−0.03	0.68	0.704
**Predictor**	**β**	**95% CI**	***t*-value**	
Noise discomfort (auditory)	−0.01	[−0.26, 0.23]	−0.09	0.929
Perceived task difficulty (auditory noise)	0.18	[−0.06, 0.42]	1.46	0.148
Motor skills	−0.06	[−0.37, 0.24]	−0.42	0.674
Executive functions	0.03	[−0.34, 0.41]	0.17	0.864
Perception	0.02	[−0.32, 0.35]	0.09	0.927
Learning	0.15	[−0.22, 0.52]	0.79	0.430
Sex (female = 1)	−0.06	[−0.50, 0.38]	−0.27	0.787
Age	−0.09	[−0.32, 0.15]	−0.75	0.459

Note. β values are standardized coefficients. CI = confidence interval. All predictors were standardized prior to analysis, and variables with skewed distributions were Yeo–Johnson transformed. N = 91.

**Table 4:** Regression results for the number of anticipatory saccades in the MGS task—visual noise benefit.

Visual Noise Benefit	R^2^	Adjusted R^2^	F(8, 83)	*p*
	0.13	0.05	**1.55**	**0.047**
**Predictor**	**β**	**95% CI**	***t*-value**	
Noise discomfort (visual)	**−0.24**	[−0.46, −0.02]	**−2.13**	**0.036**
Perceived task difficulty (visual noise)	0.17	[−0.05, 0.38]	1.57	0.120
Motor skills	**−0.30**	[−0.60, −0.00]	**−2.01**	**0.048**
Executive functions	−0.08	[−0.44, 0.29]	−0.41	0.681
Perception	0.09	[−0.23, 0.42]	0.58	0.563
Learning	0.03	[−0.34, 0.39]	0.14	0.887
Sex (female = 1)	−0.02	[−0.45, 0.41]	−0.10	0.924
Age	−0.07	[−0.29, 0.16]	−0.60	0.552

Note. β values are standardized coefficients. CI = confidence interval. Significant effects (*p* < 0.05) are shown in bold. All predictors were standardized prior to analysis, and variables with skewed distributions were Yeo–Johnson transformed. N = 92.

## Data Availability

Data will be made available upon reasonable request.

## Supplementary Materials

The following supporting information can be downloaded at: https://www.mdpi.com/article/10.3390/jemr19010018/s1, Table S1: Correlation results for the number of intrusive saccades in the prolonged fixation (PF) task.; Table S2: Correlation results for the number of intrusive saccades in the prolonged fixation (PF) task in visual noise levels 25% and 50%.; Table S3: Correlation results for the number of anticipatory saccades in the Memory Guided Saccade (MGS) task.; Table S4: Correlation results for the number of anticipatory saccades in the Memory Guided Saccade (MGS) task in visual noise levels 25% and 50%.; Table S5: Regression results for the number of intrusive saccades in the PF task—visual noise 25% benefit.; Table S6: Regression results for the number of intrusive saccades in the PF task—visual noise 50% benefit.; Table S7: Regression results for the number of anticipatory saccades in the MGS task—visual noise 25% benefit.; Table S8: Regression results for the number of anticipatory saccades in the MGS task—visual noise 50% benefit.
