# Supplementary material for: The Influence of Noise Perception and Parent-Rated Developmental Characteristics on White Noise Benefits in Children
Source: J Eye Mov Res. 2026 Feb 5;19(1):18. doi: 10.3390/jemr19010018 (PMC12921771; doi:10.3390/jemr19010018)
Supplement: Supplementary file 1 [file jemr-19-00018-s001.zip › jemr-4075721-supplementary.pdf]

Journal of
Eye Movement Research

MDPI

Supplementary Materials

The Influence of Noise Perception and Parent-Rated Developmental Characteristics on White Noise Benefits in Children

Erica Jostrup [1,*], Marcus Nyström [2], Göran B. W. Söderlund [3], Emma Claesdotter-Knutsson [1,4], Peik Gustafsson [1] and Pia Tallberg [1,4]

[1] Child and Adolescent Psychiatry, Department of Clinical Sciences, Lund University, 221 84 Lund, Sweden; emma.claesdotter-knutsson@med.lu.se (E.C.-K.); peik.gustafsson@med.lu.se (P.G.); pia.tallberg@med.lu.se (P.T.)
[2] Humanities Lab, Lund University, 221 00 Lund, Sweden; marcus.nystrom@humlab.lu.se
[3] Faculty of Teacher Education Arts and Sports, Western Norway University of Applied Sciences, 6856 Sogndal, Norway; goran.soderlund@hvl.no
[4] Outpatient Department, Child and Adolescent Psychiatry Clinic, Region Skåne, 221 85 Lund, Sweden
* Correspondence: erica.jostrup@med.lu.se

Note. Participants completed the tasks under both 25% and 50% visual noise conditions. In the main analyses, these conditions were averaged to derive a single visual noise benefit score, as prior analyses using the same dataset showed no reliable performance differences between the two visual noise levels. The 25% and 50% conditions are presented separately here for transparency and completeness.

Table S1. Correlation results for the number of intrusive saccades in the prolonged fixation (PF) task.

Predictor	Noise modality	r	*p*	N
Noise discomfort	Visual	0.331	**0.002**	90
Learning skills	Visual	-0.217	**0.042**	87
Language and communication	Visual	-0.191	0.071	90
Executive functions	Visual	-0.176	0.097	90
Social skills	Auditory	-0.165	0.117	92
Social skills	Visual	-0.155	0.147	89
Motor skills	Visual	-0.123	0.249	89
Executive functions	Auditory	-0.105	0.315	93
Memory	Visual	-0.105	0.324	90
Motor skills	Auditory	-0.104	0.325	92
Learning skills	Auditory	-0.097	0.361	90
Perception	Visual	-0.045	0.672	90
Perceived task difficulty	Visual	-0.036	0.740	90
Noise discomfort	Auditory	-0.030	0.772	93
Perception	Auditory	0.029	0.781	93
Perceived task difficulty	Auditory	-0.028	0.794	93
Language and communication	Auditory	0.025	0.816	93
Memory	Auditory	-0.010	0.925	93

Table S2. Correlation results for the number of intrusive saccades in the prolonged fixation (PF) task in visual noise levels 25% and 50%.

Predictor	Noise modality	r	*p*	N
Noise discomfort	Visual 25%	0.302	**0.004**	91
Noise discomfort	Visual 50%	0.268	**0.010**	93
Learning skills	Visual 25%	-0.198	0.0647	89
Executive functions	Visual 25%	-0.171	0.106	91
Learning skills	Visual 50%	-0.159	0.135	90
Social skills	Visual 25%	-0.155	0.144	90
Language and communication	Visual 25%	-0.143	0.177	91
Motor skills	Visual 25%	-0.140	0.189	90
Social skills	Visual 50%	-0.134	0.202	92
Language and communication	Visual 50%	-0.120	0.251	93
Executive functions	Visual 50%	-0.113	0.280	93
Memory	Visual 25%	-0.094	0.378	91
Perceived task difficulty	Visual 50%	-0.068	0.518	93
Perception	Visual 25%	-0.055	0.606	91
Motor skills	Visual 50%	-0.050	0.637	92
Memory	Visual 50%	-0.047	0.657	93
Perception	Visual 50%	0.033	0.753	93
Perceived task difficulty	Visual 25%	0.004	0.973	91

Table S3. Correlation results for the number of anticipatory saccades in the Memory Guided Saccade (MGS) task.

Predictor	Noise modality	r	*p*	N
Motor skills	Visual	-0.256	**0.012**	95
Perceived task difficulty	Auditory	0.198	0.055	95
Learning skills	Visual	-0.166	0.111	93
Perception	Visual	-0.160	0.120	96
Noise discomfort	Visual	-0.159	0.122	96
Memory	Visual	-0.155	0.132	96
Executive functions	Visual	-0.150	0.145	96
Noise discomfort	Auditory	0.140	0.175	95
Language and communication	Auditory	0.130	0.208	95
Learning skills	Auditory	0.129	0.219	92
Executive functions	Auditory	0.116	0.265	95
Language and communication	Visual	-0.115	0.266	96
Memory	Auditory	0.103	0.320	95
Perceived task difficulty	Visual	0.093	0.366	96
Perception	Auditory	0.059	0.571	95
Social skills	Visual	-0.054	0.606	95
Social skills	Auditory	0.051	0.629	94
Motor skills	Auditory	0.033	0.751	94

Table S4. Correlation results for the number of anticipatory saccades in the Memory Guided Saccade (MGS) task in visual noise levels 25% and 50%.

Predictor	Noise modality	r	*p*	N
Motor skills	Visual 50%	-0.227	**0.027**	95
Noise discomfort	Visual 25%	-0.196	0.055	96
Motor skills	Visual 25%	-0.185	0.073	95
Learning skills	Visual 25%	-0.167	0.109	93
Perceived task difficulty	Visual 50%	0.146	0.155	96
Perception	Visual 50%	-0.136	0.185	96
Memory	Visual 50%	-0.133	0.197	96
Executive functions	Visual 50%	-0.124	0.230	96
Perception	Visual 25%	-0.123	0.234	96
Executive functions	Visual 25%	-0.121	0.242	96
Memory	Visual 25%	-0.118	0.252	96
Learning skills	Visual 50%	-0.109	0.296	93
Language and communication	Visual 50%	-0.099	0.338	96
Social skills	Visual 25%	-0.092	0.378	95
Language and communication	Visual 25%	-0.087	0.400	96
Noise discomfort	Visual 50%	-0.076	0.462	96
Perceived task difficulty	Visual 25%	-0.011	0.914	96
Social skills	Visual 50%	-0.005	0.961	95

Note. Significant effects ($p < 0.05$) are shown in bold.

Table S5. Regression results for the number of intrusive saccades in the PF task – visual noise 25% benefit.

Visual noise 25% benefit	R^2	Adjusted R^2	F(8, 78)	*p*
	0.16	0.07	1.83	0.083
**Predictor**	**β**	**95% CI**	**t-value**	
Noise discomfort (visual 25%)	**0.33**	[0.10, 0.56]	**2.86**	**0.006**
Perceived task difficulty (visual noise)	–0.12	[–0.34, 0.10]	-1.09	0.279
Motor skills	–0.08	[–0.39, 0.23]	-0.51	0.613
Executive functions	–0.01	[–0.37, 0.34]	-0.08	0.938
Perception	0.15	[–0.18, 0.48]	0.90	0.373
Learning	–0.26	[–0.63, 0.11]	-1.41	0.162
Sex (female = 1)	0.09	[–0.36, 0.53]	0.38	0.703
Age	0.07	[–0.16, 0.30]	0.60	0.550

Note. β values are standardized coefficients. CI = confidence interval. Significant effects ($p < 0.05$) are shown in bold. All predictors were standardized prior to analysis, and variables with skewed distributions were Yeo–Johnson transformed. N = 87.

Table S6. Regression results for the number of intrusive saccades in the PF task – visual noise 50% benefit.

Visual noise 50% benefit	R^2	Adjusted R^2	F(8, 80)	*p*
	0.18	0.10	**2.19**	**0.037**
**Predictor**	**β**	**95% CI**	**t-value**	
Noise discomfort (visual 50%)	**0.33**	[0.11, 0.55]	**2.97**	**0.004**
Perceived task difficulty (visual noise)	–0.17	[–0.39, 0.05]	-1.57	0.120
Motor skills	–0.04	[–0.33, 0.26]	-0.24	0.812
Executive functions	–0.02	[–0.37, 0.33]	-0.12	0.908
Perception	0.27	[–0.05, 0.58]	1.68	0.097
Learning	–0.34	[–0.69, 0.02]	-1.89	0.062
Sex (female = 1)	0.32	[–0.12, 0.75]	1.45	0.152
Age	0.07	[–0.16, 0.30]	0.64	0.525

Note. β values are standardized coefficients. CI = confidence interval. Significant effects ($p < 0.05$) are shown in bold. All predictors were standardized prior to analysis, and variables with skewed distributions were Yeo–Johnson transformed. N = 89.

Table S7. Regression results for the number of anticipatory saccades in the MGS task – visual noise 25% benefit.

Visual noise 25% benefit	R^2	Adjusted R^2	F(8, 83)	*p*
	0.12	0.03	1.40	0.211
**Predictor**	**β**	**95 % CI**	**t-value**	
Noise discomfort (visual 25 %)	**–0.27**	[–0.49, –0.05]	**-2.427**	**0.017**
Perceived task difficulty (visual noise)	0.04	[–0.17, 0.26]	0.389	0.699
Motor skills	–0.17	[–0.47, 0.13]	-1.107	0.272
Executive functions	0.02	[–0.35, 0.38]	0.105	0.917
Perception	0.09	[–0.23, 0.42]	0.580	0.563
Learning	–0.11	[–0.47, 0.26]	-0.585	0.560
Sex (female = 1)	–0.32	[–0.75, 0.11]	-1.469	0.146
Age	0.06	[–0.17, 0.28]	0.487	0.627

Note. β values are standardized coefficients. CI = confidence interval. Significant effects ($p < 0.05$) are shown in bold. All predictors were standardized prior to analysis, and variables with skewed distributions were Yeo–Johnson transformed. N = 92.

Table S8. Regression results for the number of anticipatory saccades in the MGS task – visual noise 50% benefit.

Visual noise 50% benefit	R^2	Adjusted R^2	F(8, 83)	*p*
	0.12	0.04	1.41	0.204
**Predictor**	**β**	**95 % CI**	**t-value**	
Noise discomfort (visual 50 %)	–0.12	[–0.35, 0.10]	-1.107	0.272
Perceived task difficulty (visual noise)	0.12	[–0.09, 0.34]	1.124	0.264
Motor skills	**–0.38**	[–0.68, –0.07]	**-2.469**	**0.016**
Executive functions	–0.09	[–0.46, 0.28]	-0.509	0.612
Perception	0.09	[–0.24, 0.42]	0.555	0.580
Learning	0.11	[–0.26, 0.48]	0.590	0.557
Sex (female = 1)	0.15	[–0.29, 0.59]	0.684	0.496
Age	–0.12	[–0.35, 0.11]	-1.045	0.299

Note. β values are standardized coefficients. CI = confidence interval. Significant effects ($p < 0.05$) are shown in bold. All predictors were standardized prior to analysis, and variables with skewed distributions were Yeo–Johnson transformed. N = 92.
